# Supplementary material for: Social and Spatial Clustering of People at Humanity’s Largest Gathering
Source: PLoS One. 2016 Jun 3;11(6):e0156794. doi: 10.1371/journal.pone.0156794 (PMC4892527; doi:10.1371/journal.pone.0156794)
Supplement: S1 Text — Extended discussion of how some measures of homophily can be susceptible to confounding with the size of the subgroup. The names and corresponding market shares of the 23 mainland states of India is listed. Some intuition for how censoring takes effect is also included. (PDF) [file pone.0156794.s001.pdf]

Supplementary Information for "Social and Spatial Clustering of People at Humanity's Largest Gathering"

Ian Barnett,[1] Tarun Khanna,[2] Jukka-Pekka Onnela[1*]

[1]Department of Biostatistics, Harvard University,
677 Huntington Avenue, Boston, MA 02115, USA
[2]Harvard Business School, Boston, MA 02163, USA
*To whom correspondence should be addressed; E-mail: onnela@hsph.harvard.edu.

Overview: Selecting the social homophily statistic

When comparing social homophily across the 23 states of India, care must be put into the selection of the appropriate test statistic. It is essential to select a test statistic that, holding social homophily constant, does not scale with a state's representation. We discuss two test statistics that fail to meet this criterion and show how these statistics provide motivation for the model we ultimately use.

Statistic 1: Fraction of total ties that stay within a state

The measure of social homophily considered in [1, 2] defines homophily as the fraction of total ties from a state that stay within the same state, but due to measuring absolute differences instead of relative differences, this is biased unfairly against states with small representation. Consider the following example:

Let N be the total number of nodes, W_k be the fraction of nodes in state k, D_k be the average number of neighbors of nodes in state k that are from different states, and S_k be the average number of neighbors of nodes in state k that belong to the same state. The measure of homophily considered in [1, 2] defines homophily as the fraction of total ties that are within the same state. This measure is normalized under a null model that ties are formed without regard to state membership. This measure is called inbreeding homophily and is measured as $\frac{H_k - W_k}{1 - W_k}$ where where $H_k = S_k/(S_k + D_k)$. When the alternative hypothesis is true, as is the case here where each state demonstrates significant social homophily, then inbreeding homophily lacks good interpretation and comparability between states of different sizes.

For example, consider the Delhi region, which has a $W_k = 4.1\%$. Therefore under the null hypothesis of indiscriminate pairing, we would expect the fraction of edges from the Delhi block that stay between people from the Delhi block (H_k) to be 4.1%. Instead, this observed measure is 36.2%, about 9-fold greater than what is expected under the null and showing significant social homophily. Compare this to the Jammu and Kashmir region, which has very low representation of $W_k = 0.019\%$. Under indiscriminate pairing we would expect only 0.019% of the total edges from people from the Jammu and Kashmir region to stay between people from that region, however the observed measure is 15.3%. Despite this being 805-fold more than what we would expect under the null hypothesis, the inbreeding homophily measure for Jammu and Kashmir is a mere 0.153, whereas that same measure for Delhi is 0.335 and would lead one to believe that Delhi exhibits greater homophily. In this case, looking at H_k relative to W_k to evaluate social homophily leads to qualitatively different conclusions when looked at on the difference scale as opposed to the ratio scale. The fact that the scale alone can so drastically alter one's conclusion makes the statistic untrustworthy for our purposes.

Statistic 2: Stochastic Block Model

A standard stochastic block model approach[3], assumes an equal likelihood of forming network edges between nodes in the same state. The Kumbh social network is separated into 23 blocks, one for each state. The edge probabilities for within-block edges are p_{kk}, $k = 1, \ldots, 23$, one for each of the state. For the kth block, let $A_{ij}^{(k)} = 1$ if nodes i and j are connected by an edge, and $A_{ij}^{(k)} = 0$ otherwise, for $i \neq j \in \{1, \ldots, n_k\}$ where n_k is the number of nodes in block k. The p_{kk} are calculate as:

$$p_{kk} = \frac{\sum_{i=1}^{n_k-1} \sum_{j=i+1}^{n_k} A_{ij}^{(k)}}{n_k(n_k-1)/2}$$

The p_{kk} offer a natural comparison of social homophily across the different states. Large values of p_{kk} indicate strong social homophily between individuals in block k, and small values of p_{kk} indicate weak social homophily. In Figure S1, there appears to be a strong negative association between state representation at the Kumbh and social homophily. Though this seems like compelling evidence, it should not be trusted due to its high sensitivity to model mispecification.

The model assumption of the standard stochastic block model used here is that each pair of individuals within the same state have the same probability of being socially connected. The true model is likely more complicated, as states are likely composed of numerous independent friendship groups. Consider the fictitious example presented in Figure S2. In this case, the edge probability within a friendship group is assumed to be 0.20, whereas the edge probability between two people from different friendship groups is lower at 0.04. In this example, we compare two states, state A and state B. State A is composed of four friendship groups of equal size, and state B is composed of only two such groups. As demonstrated in Figure S2, despite having the same within-group edge probability of 0.20 and the same between-group edge probability of 0.04, and therefore the same degree of social homophily, the average overall edge probability is twice as large in state B than it is in state A. This average overall edge probability is equivalent to the p_{kk} measured by the stochastic block model. As a result, states with greater representaiton at the Kumbh will have larger values of p_{kk} even if the they are no more homophilous than states with less representation.

Though friendship groups within state are unobserved, if this internal block structure is ignored, Figure S2 makes it clear that strong biases can emerge when comparing p_{kk} between states with different size representation at the Kumbh. To avoid this issue we can restrict the analysis to within-friendship group pairs of nodes, ignoring the between-friendship group pairs. In other words, we target only the network ties in the blue friendhsip groups of Figure S2 and ignore the red regions. Unfortunately, these friendship groups are unobserved. We circumvent this missing information by restricting to connected triangles under the assumption that if a pair of nodes is a maximum distance of 2 edges apart from each other, then they both belong to the same friendship group. Under this approach, the situation in S2 would correctly estimate that both states A and B have the same strength of social homophily.

Estimating the proportion of days customers do not use their phone at the Kumbh.

Customers are only observed when they use their phone. This introduces a potentially large missing data component which can bias attendance estimates downwards. When estimating the daily attendance at the Kumbh, unless the missing data is properly accounted for, the customers who do not use their phone on a particular day will bias attendance downwards for that day (Figure S3). With some assumptions on the nature of the missing data, this bias can be adjusted for. Assume that each customer uses their phone on a day with probability p, independently for each day. If there were no censoring, then we could estimate p by summing the number of cell phones used each day across the full 90 day period ($\sum_{d=1}^{90} \sum_{r=1}^{23} N_{rd}$) and then divide that by the cumulative number of unique individuals (C) times their average length of stay ($\bar{L} = 18.1$ days).

To adjust for censoring, the number of censored days prior to the first phone usage follows a geometric distribution with parameter p. This modeling assumption holds if the likelihood of phone usage on each day is independent with equal probability. The geometric mean is $(1-p)/p$, which can be interpreted as the expected number of censored days where a person comes to the Kumbh but does not use their phone and so is unobserved for those days. The same applies to the censored days after the final phone usage, so in total there are an expected $2(1-p)/p$ unobserved days for

each individual where they were at the Kumbh but did not use their phone. This leads to the following estimate of p:

$$\hat{p} = \frac{\sum_{d=1}^{90} \sum_{r=1}^{23} N_{rd}}{C \cdot \bar{L} + C \cdot 2 \cdot \frac{1-\hat{p}}{\hat{p}}}$$

Solving for $\hat{p}$ yields:

$$\hat{p} = \frac{\sum_{d=1}^{90} \sum_{r=1}^{23} N_{rd} - 2 \cdot C}{\bar{L} \cdot C - 2 \cdot C}$$

With $C = 4,538,652$ and $\sum_{d=1}^{90} \sum_{r=1}^{23} N_{rd} = 38,629,454$ we reach a final estimate of $\hat{p} = \mathbf{40.4}\%$. Without adjusting for censoring, the estimated probability rises to 47.0%.

Attendance estimates and the corresponding confidence intervals.

Though the CDR data allows us to estimate daily and cumulative cell phone usage, we need to extrapolate by market share, proportion of wireless subscribers in India ($V = 71.3\%$ in 2013), as well correct for unobserved individuals who do not use their phones in order to arrive at estimates for the total daily and cumulative attendance at the Kumbh. Let U be the proportion of observed customers, i.e. the cumulative proportion of customers that attended the Kumbh and used their phone at least once over the three month period from January 1st to March 31st. Let M_r be the market share of Bhart Airtel in state r during the first quarter of 2013 (listed in Table S1). Recalling the $\hat{p} = 0.404$ from the preivous section, we estimate the total predicted attendance of the Kumbh on day d, P_d, as:

$$\hat{P}_d = \sum_{r=1}^{23} \frac{N_{rd}}{\hat{p} \cdot \hat{M}_r \cdot \hat{V} \cdot U}$$

To construct a confidence interval we condition on the N_{rd} and the cumulative attendance C. The three random variables, $\hat{V}$, $\hat{M}_r$, and $\hat{p}$, contribute to the variability. We assume each $\hat{M}_r$ is a binomial proportion with size N_{rd} and probability M_r. With 1.27 billion people in India in 2013, we assume $\hat{V}$ is a binomial proportion with size $1,270,000,000$ and probability $V = 0.713$. The only random component of $\hat{p}$ is $\bar{L} \sim N(18.1, 26.2^2/C) = N(18.1, 1.51 \cdot 10^{-4})$, where normality follows from the central limit theorem. Thus, in order to generate random instances of $\hat{P}_d$ we need only generate a random instance of $\bar{L}$, $\hat{M}_r$, and $\hat{V}$. This is repeated $1,000$ times and the 2.5% and 97.5% quantiles represent the 95% confidence bounds. Unfortunately we have no means of quantifying uncertainty in U, which is why we instead provide the sensitivity to this parameter in panel C of Figure 2 in the main text.

For estimating cumulative attendance, the same extrapolations are used except $\hat{M}_r$ no longer applies, because so long as a person uses their phone at least once it doesn't matter what proportion of the days at the Kumbh their phone is inactive. For that reason the cumulative estimate of the lower bound on January 1st is actually lower than the daily estimate of the lower bound, because the daily estimate is divided by the $\hat{M}_r$. This discrepancy is evident when comparing January first in panels A and B of Figure 2 of the main text.

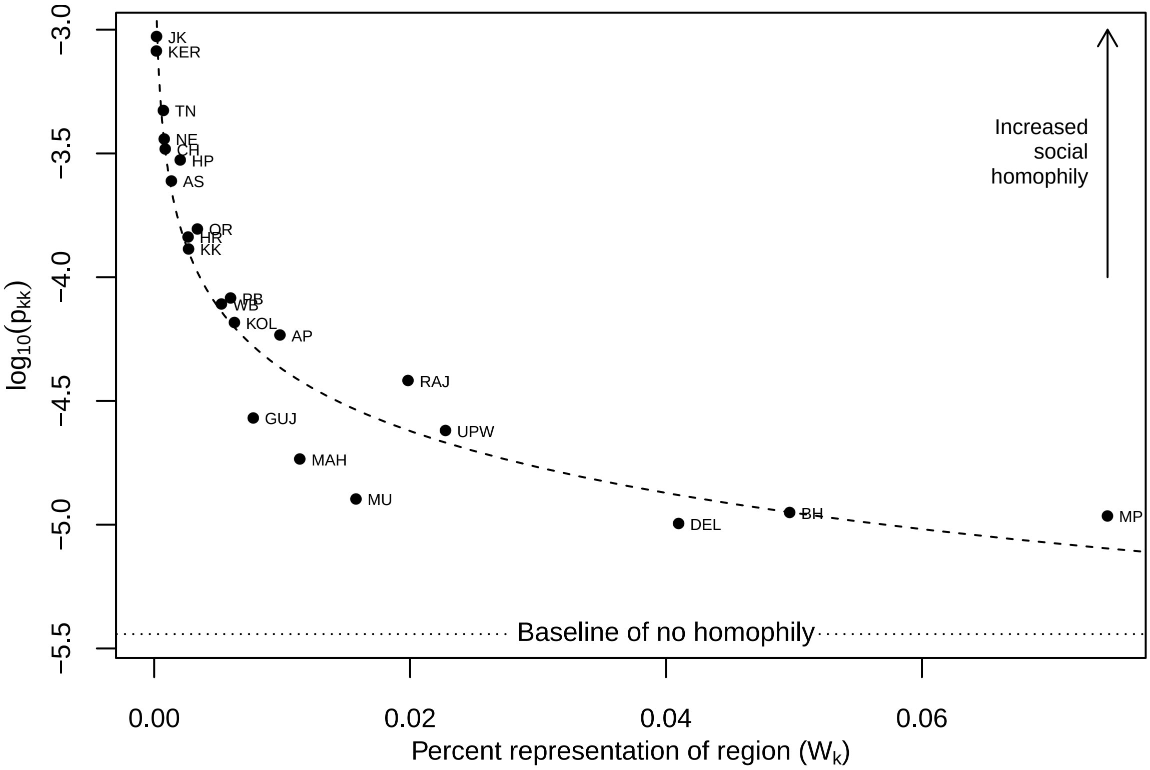

Fig. S1 Stochastic block model edge probabilities by state. The p_{kk} represent the probability that any random two nodes in state k share an edge, assuming this probability is the same for all pairs of nodes in state k. The strong association between this probability and state representation is heavily biased under model mispecification as is more likely the case here, exagerating the result. The baseline probability is calculated assuming no block structure, i.e. all nodes have the same probability of being connected to one another regardless of state membership.

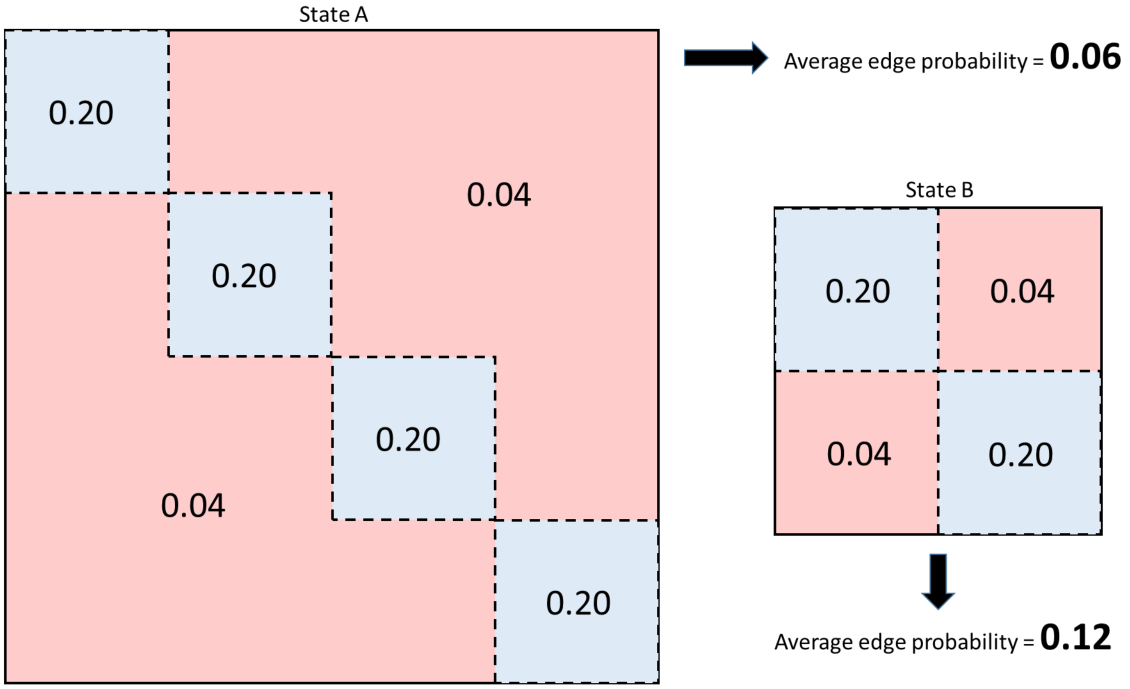

Fig. S2 Simple illustration of the bias produced by the stochastic block model under model misspecification. Social groups are displayed in blue, and are assumed to all be of equal size. The probability that two people in the same social group share an edge is 0.20. The probability that two people in different social groups share an edge is 0.04. States A and B are constructed to have identical homophily, i.e. the probability of an edge between two people in the same social group is the same for both states. The average edge probability displayed takes the average over all possible pairs of nodes in the state.

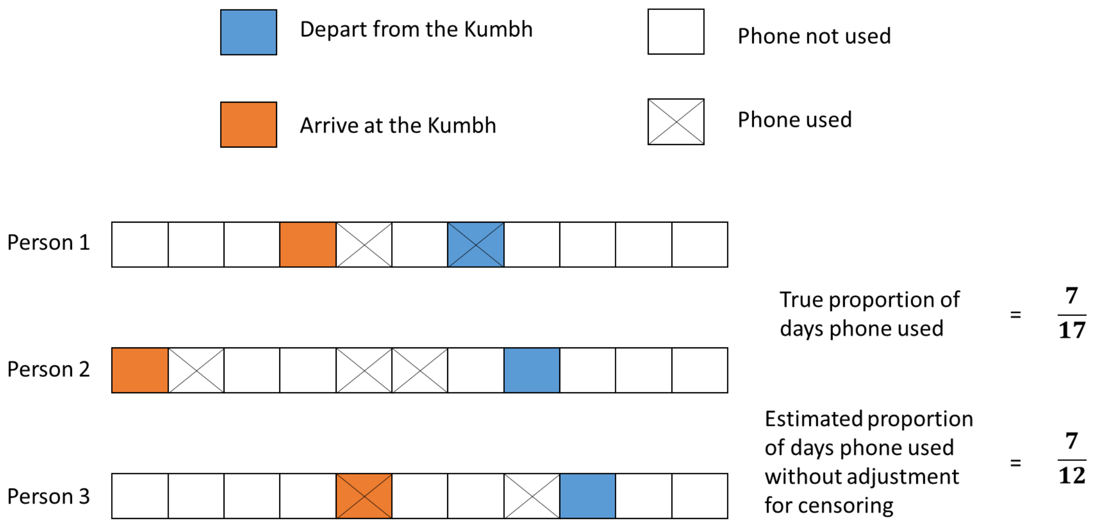

Fig. S3 Schematic for estimation of the probability of phone usage on any given day. Each square represents a different day, and it is assumed that a person arrives at and departs from the Kumbh only once. The estimated proportion of days a phone is used is calculated as the total number days a phone is used summed across all customers, divided by the length of stay summed across all customers.

Table S1 State Acronyms and Bharti Airtel market share. The acronyms for the twenty-three telecommunications states in India used by Bharti Airtel are listed. In addition, the market share of Airtel as measured by the percentage of the total number of people in the state with some form of subscription to a phone plan, taken from the month of January 2013.

State Name	State Acronym	Airtel Market Share
Karnataka	KK	42.6%
Bihar	BH	40.8%
Rajasthan	RAJ	39.7%
Jammu and Kashmir	JK	38.5%
Orissa	OR	36.5%
Himachal Pradesh	HP	36.2%
Andhra Pradesh	AP	36.1%
Assam	AS	34.6%
North East	NE	33.6%
Delhi	DEL	32.9%
Chennai	CH	32.6%
Punjab	PB	30.9%
West Bengal	WB	28.2%
Madhya Pradesh	MP	28.1%
Uttar Pradesh East	UPE	26.2%
Kolkata	KOL	25.0%
Tamil Nadu	TN	20.4%
Maharashtra	MAH	19.0%
Uttar Pradesh West	UPW	17.5%
Gujarat	GUJ	17.2%
Mumbai	MU	16.5%
Haryana	HR	15.7%
Kerala	KER	13.7%

References

[1] J. S. Coleman, *Human Organization* **17**, 28 (1958).

[2] S. Currarini, M. O. Jackson, P. Pin, *Econometrica* **77**, 1003 (2009).

[3] P. W. Holland, K. B. Laskey, S. Leinhardt, *Social networks* **5**, 109 (1983).
